# Cultural Affordances: Scaffolding Local Worlds Through Shared Intentionality and Regimes of Attention

**DOI:** 10.3389/fpsyg.2016.01090

**Published:** 2016-07-26

**Authors:** Maxwell J. D. Ramstead, Samuel P. L. Veissière, Laurence J. Kirmayer

**Affiliations:** ^1^Department of Philosophy, McGill University, Montreal, QCCanada; ^2^Division of Social and Transcultural Psychiatry, Department of Psychiatry, McGill University, Montreal, QCCanada; ^3^Department of Anthropology, McGill University, Montreal, QCCanada; ^4^Raz Lab in Cognitive Neuroscience, McGill University, Montreal, QCCanada; ^5^Department of Communication and Media Studies, Faculty of Humanities, University of JohannesburgJohannesburg, South Africa

**Keywords:** affordances (ecological psychology), cultural affordances, radical embodied cognition, enactive cognitive neuroscience, free-energy principle, predictive processing, regimes of attention, cognitive anthropology

## Abstract

In this paper we outline a framework for the study of the mechanisms involved in the engagement of human agents with cultural affordances. Our aim is to better understand how culture and context interact with human biology to shape human behavior, cognition, and experience. We attempt to integrate several related approaches in the study of the embodied, cognitive, and affective substrates of sociality and culture and the sociocultural scaffolding of experience. The integrative framework we propose bridges cognitive and social sciences to provide (i) an expanded concept of ‘affordance’ that extends to sociocultural forms of life, and (ii) a multilevel account of the socioculturally scaffolded forms of affordance learning and the transmission of affordances in patterned sociocultural practices and regimes of shared attention. This framework provides an account of how cultural content and normative practices are built on a foundation of contentless basic mental processes that acquire content through immersive participation of the agent in social practices that regulate joint attention and shared intentionality.

## Introduction

The acquisition of culture is notoriously difficult to study. Over 70 years of research on the development of person-perception, for example, have made it clear that children as young as 4 years of age have already acquired implicit biases about ethnicity and other socially constructed categories of persons (Clark and Clark, 1939; Clark, 1963; Hirschfeld, 1996; Machery and Faucher, 2005; Aboud and Amato, 2008; Kelly et al., 2010; Huneman and Machery, 2015; Pauker et al., 2016). These biases are consistent with the dominant culture of their societies, but are most often not consciously held or explicitly taught by their caregivers and educators. While most young children express a positive bias toward people they identify as members of their own group, children from minority groups typically show preferences for dominant groups, rather than for persons of their own ethnicity (Clark and Clark, 1939; Kinzler and Spelke, 2011). How such biases are acquired is still an open question. Ethnographic studies of socialization, education, and language acquisition have pointed to broad cross-cultural variations in how children are instructed, spoken to, expected to behave, involved in community activities, and exposed to other socializing agents beyond nuclear or extended families (Mead, 1975; Schieffelin and Ochs, 1999; Rogoff, 2003). However, by age 5, children across cultures have for the most part become proficient in the dominant set of expectations and representations of their cultures, despite the much discussed poverty of cultural stimuli to which they are exposed (Chomsky, 1965). These matters point to a human propensity for ‘picking up’ the broad scripts of culture even without any explicit instruction. In other words, we all come to acquire the shared background knowledge, conceptual frameworks, and dominant values of our culture. The presence of intuitive or implicit, yet stable and widely shared beliefs and attitudes among children constitutes a challenging problem for cognitive and social science.

In this paper, we outline a framework for the study of the mechanisms that mediate the acquisition of cultural knowledge, values, and practices in terms of perceptual and behavioral *affordances*. Our aim is to better understand how culture and context shape human behavior and experience by integrating several related approaches in the study of the embodied, cognitive, and affective substrates of action and the sociocultural scaffolding of embodied experience. The integrative framework we propose bridges cognitive and social sciences to provide (i) an expanded concept of ‘affordance’ that extends to sociocultural forms of life, and (ii) a multilevel account of the socioculturally scaffolded forms of affordance learning and the transmission of affordances in patterned sociocultural practices.

The context of the present discussion is the search for the ‘natural origins of content’ (Hutto and Satne, 2015). We hope to contribute to the naturalistic account of the emergence of semantic content, that is, of the evolution (in phylogeny) and acquisition (in ontogeny) of representational or propositional content. Cultural worlds seem to be full of meaningful ‘content’—of explicit ways to think about and respond to the world in terms of kinds of agents, actions, and salient events. ‘Content,’ here, is defined in terms of representational relations with satisfaction conditions: a vehicle *x* bears some semantic or representational content *y* just in case there are satisfaction conditions which, when they obtain, tell us that the vehicle is *about* something. Semantics is an intensional notion (Millikan, 1984, 2004, 2005; Haugeland, 1990; Piccinini, 2015). How do humans acquire this cultural knowledge and capacity to respond in social contexts in ways that actors and others find meaningful and appropriate?

We hypothesize that agents acquire semantic content through their immersion in, and dynamic engagement with, feedback or looping mechanisms that mediate shared intentionality and shared attention. Semantic content, we suggest, is realized in culturally shared expectations, which are embodied at various levels (in brain networks, cultural artifacts, and constructed environments) and are enacted in ‘regimes’ of shared attention. We generalize contemporary ecological, affordance-based models of cognitive systems adapting to their contexts over ontogeny and phylogeny to account for the acquisition of cultural meanings and for the elaborate scaffoldings constituted by constructed, ‘designer’ niches (Hutchins, 2014; Kirchhoff, 2015a; Clark, 2016). We suggest that ‘regimes of shared attention’—that is, patterned cultural practices (Roepstorff et al., 2010) that direct the attention of participant agents—modulate the acquisition of culturally specific sets of expectations. Recent work in computational neuroscience on predictive processing provides a model of how cultural affordances could scaffold the acquisition of socially shared representational content. In what follows, we shall sketch a multilevel framework that links neural computation, embodied experience, cultural affordances, and the social distribution of representations.

We begin by specifying a conceptual framework for ‘cultural affordances’, building on recent accounts of the notion of affordances in ecological, enactivist, and radical embodied cognitive science (**Box 1**). We propose to distinguish two kinds of cultural affordances: ‘natural’ affordances and ‘conventional’ affordances. Natural affordances are possibilities for action, the engagement with which depends on an organism or agent exploiting or leveraging reliable correlations in its environment with its set of abilities. For instance, given a human agent’s bipedal phenotype and related ability to walk, an unpaved road affords a trek. Conventional affordances are possibilities for action, the engagement with which depends on agents’ skillfully leveraging explicit or implicit expectations, norms, conventions, and cooperative social practices. Engagement with these affordances requires that agents have the ability to correctly infer (implicitly or explicitly) the culturally specific sets of expectations in which they are immersed—expectations about how to interpret other agents, and the symbolically and linguistically mediated social world. Thus, a red light affords stopping not merely because red lights correlate with stopping behavior, but also because of shared (in this case, mostly explicit) norms, conventions, and rules. Both kinds of cultural affordances are relevant to understanding human social niches; and both natural and conventional affordances may be socially constructed, albeit in different ways (Hacking, 1999). Human biology is cultural biology; culture has roots in human biological capacities. The affordances with which human beings engage are cultural affordances.

**BOX 1. Basic concepts of a framework for cultural affordances**

**Affordance**: A relation between a feature or aspect of organisms’ material environment and an ability available in their form of life (Chemero, 2003, 2009; Bruineberg and Rietveld, 2014; Rietveld and Kiverstein, 2014).

**Landscape of affordances**: The total ensemble of available affordances for a population in a given environment. This landscape corresponds to what evolutionary theorists in biology and anthropology call a ‘niche’ (Rietveld, 2008a,c; Rietveld et al., 2013; Bruineberg and Rietveld, 2014; Rietveld and Kiverstein, 2014).

**Field of affordances**: Those affordances in the landscape with which the organism, as an autonomous individual agent, dynamically copes and intelligently adapts. The field refers to those affordances that actually engage the individual organism because they are salient at a given time, as a function of the interests, concerns, and states of the organism (Rietveld, 2008a,c; Bruineberg and Rietveld, 2014; Rietveld and Kiverstein, 2014).

**Cultural affordance**: The kind of affordance that humans encounter in the niches that they constitute. There are two kinds of cultural affordances: natural and conventional affordances.

**Natural affordance**: Possibilities for action (i.e. affordances), the engagement with which depends on the exploitation or leveraging by an organism of ‘natural information’, that is, reliable correlations in its environment, using its set of phenotypical and encultured abilities (roughly what Grice meant by ‘natural meaning’) (Piccinini and Scarantino, 2011; Piccinini, 2015).

**Conventional affordance**: Possibilities for action, the engagement with which depends on agents’ skillfully leveraging explicit or implicit expectations, norms, conventions, and cooperative social practices in their ability to correctly infer (implicitly or explicitly) the culturally specific sets of expectations of which they are immersed. These are expectations about how to interpret other agents, and the symbolically and linguistically mediated social world (Scarantino and Piccinini, 2010; Tomasello, 2014; Satne, 2015; Scarantino, 2015).

We then assess the tensions between our proposed framework and radical enactivist and embodied approaches, which are typically committed to forms of non- (or even anti-) representationalism. On these views, perception, cognition, and action need not involve computational or representational resources. The scope of this claim varies. For some, this entails a rejection of computational or representational models and metaphors in the study of the mind—a staunch commitment to anti-representationalism (Varela et al., 1991; Gallagher, 2001, 2008; Thompson, 2007; Chemero, 2009). More conciliatory positions instead suggest that basic cognitive processes are without content, but accommodate a place for contentful cognition. They claim that certain typically human forms of cognition involve representations, in the sense that human agents have the dispositions (mechanisms, behavioral repertoires, etc.) that are required to immersively engage with sociocultural content (e.g., patterned symbolic practices, linguistic constructions, storytelling and narration). We argue that contemporary computational neuroscience complements the more conciliatory of these approaches by providing minimal neural-computational scaffolding for the skilled engagement of organisms with the available affordances.

Having done this, we turn to affordances in social and linguistic forms of life. We examine local ontologies, understood as sets of shared expectations, as well as the complex feedback relations (or looping effects) between these ontologies and human modes of communication, shared intentionality, and shared attention. Drawing on the skilled intentionality framework (Bruineberg and Rietveld, 2014), we examine the dynamics of cultural affordance acquisition through patterned cultural practices, notably attentional practices. We hypothesize that feedback mechanism between patterned regimes of attention and shared forms of intentionality (notably shared expectations and immersion in local ontologies) leads to the acquisition of such affordances. This framework can guide future research on multilevel, recursive, nested cultural affordances and the social norms and individual expectations on which they depend.

## A Theoretical Framework for Affordances

Much recent work in cognitive science has been influenced by the notion of affordances originally introduced by Gibson (1986). The interdisciplinary framework currently being developed to study affordances provides us with a point of departure for thinking about the evolution and acquisition of semantic, representational content. The aim of this section is to clarify the implications of adopting this framework.

Affordances are central to the emerging ‘enactivist’ and ‘radical embodied’ paradigms in cognitive neuroscience. Theorists of enactive cognition model the intelligent adaptive behavior of living cognitive systems as the dynamic constitution of meaning and salience in rolling cycles of perception and action, explicitly recognizing the emergence of meaning and salience in the active, embodied engagement of organisms with their environment (Di Paolo, 2005, 2009; Noë, 2005; Thompson, 2007; Froese and Di Paolo, 2011; Hutto and Myin, 2013; Di Paolo and Thompson, 2014; Hutto and Satne, 2015; Kirchhoff, 2016). Embodied approaches in cognitive science explain the feats of intelligence displayed by cognitive systems by considering the dependence of cognition on the various aspects of the body as it engages with its environment, both internal and external (Barsalou, 2008; Shapiro, 2010). ‘Radical embodied’ cognitive science extends the theoretical framework of ecological psychology (Gibson, 1986) to the embodied cognition paradigm, providing a phenomenologically plausible account of active, dynamical coping (Thompson and Varela, 2001; Chemero, 2003, 2009; Bruineberg and Rietveld, 2014; Rietveld and Kiverstein, 2014). Recently, the enactive, radical enactive, and radical embodied approaches have been extended to ‘higher-order’ social and cultural systems (Froese and Di Paolo, 2011; Hutto and Myin, 2013; Rietveld and Kiverstein, 2014). This latter branch of enactivist theory will concern us especially.

### Perspectives, Affordances, and Phenomenology

One of the distinctive contributions of ecological, radical embodied, and enactivist theories of cognition is their shared emphasis on the point of the view of the organism itself, understood as an intentional center of meaningful behavior. The implication of these ‘perspectivist’ approaches in cognitive science is that the world is disclosed as a set of ‘affordances,’ that is, possibilities for action afforded to organisms by the things and creatures that populate its environmental niche, as engaged through their perceptual and sensorimotor abilities (Turvey et al., 1981; Turvey, 1992; Reed, 1996; Heft, 2001; Silva et al., 2013; cf. also Varela, 1999; Thompson, 2007). To paraphrase Wittgenstein, the world is the totality of possibilities of action, not of things. Perspectivist approaches in cognitive science operationalize this view of the organism and propose an account of perception, cognition, and action that is closer to the phenomenology of everyday experience.

Affordances provide an alternative framework for thinking about perception, cognition, and action that dissolves the strict conceptual boundary between these categories in a way that is closer to the phenomenology of everyday life^1^. This approach echoes the kernel insights of the phenomenology of Heidegger (1927/1962) and Merleau-Ponty (1945/2012, 1964/1968) about perception and action. Cognitive agents experience the world perceptually through the mediation of action, as a function of those actions that things in the world afford. For example, my cup of coffee is not first perceived as having such and such properties (size, shape, color), and only then as providing the opportunity for sipping dark roast. Instead, my filled cup is directly perceived as affording the action of sipping. Filled cups of coffee afford sipping; a paved road affords walking; a red traffic light affords stopping. The claim, then, is that cognitive agents typically do not encounter the world that they inhabit as a ‘pre-given,’ objective, action-neutral set of things and properties, to be reconstructed in perception and cognition on the basis of sensory information, as classical models in cognitive science once suggested (e.g., Fodor, 1975; Marr, 1982; Dawson, 2013). The things that we engage are disclosed instead directly as opportunities for action—that is, as affordances. As Heidegger (1927/1962) famously argued, it is only when my smooth coping breaks down (say, when I run out of coffee, or when the cup breaks) that the objective properties of the cup become salient, present in perceptual experience at all.

The principal motivation for thinking of perception, cognition, and action in terms of engagement with affordances is that cognitive scientific accounts of these activities ought to be coherent with the phenomenology of action and perception in everyday life. Phenomenology tells us that there are dense interrelations between action and perception, that perception is mainly about the control of action, and that action serves to guide perception (Merleau-Ponty, 1945/2012, 1964/1968). Affordances provide a framework apt for this task, allowing us to integrate phenomenological experience into our models of explanation in cognitive science (Varela, 1996; Petitot et al., 1999). As the story goes, in the wake of the behaviorist turn, experiential factors and mentalist language were banished from psychology (Watson, 1913; Skinner, 2011). Cognitive science rehabilitated mentalism, at least to some extent, in its postulation of cognitive states and processes (Fodor, 1975; Putnam, 1975). Most contemporary functionalist and mechanistic accounts of cognition, however, contend that it is possible to exhaustively explain a cognitive function by specifying its functional organization or the mechanism that implements that function (e.g., Craver, 2007; Bechtel, 2008). As we shall see presently, the perspectivist emphasis on the dynamics of the phenomenology of everyday life that characterizes enactive and ecological approaches allows us to account for cognitive functions with a conceptual framework that explicitly bridges the phenomenology of action and perception, system dynamics, and functionalist cognitive neuroscience.

### Landscapes and Fields

Affordances, as possibilities for action, are fundamentally interactional. Their existence depends both on the objective material features of the environment and on the abilities of different kinds of organisms. This dependence on interaction does not mean that affordances have no objective reality or generalizability (Chemero, 2003, 2009). Affordances exist independently of specific individual organisms. Their existence is relative to sets of abilities available to certain kinds of organisms in a given niche. ‘Abilities,’ here, refers to organisms’ or agents’ capabilities to skillfully engage the environment, that is, to adaptively modulate its patterns of action-perception to couple adaptively to the environment. Without certain abilities, correlative opportunities for action are unavailable. Certain chimpanzees, for instance, are able to use rocks to cracks nuts. But for nuts and rocks to afford cracking, the chimp must already be cognitively and physiologically equipped for nut-cracking. In Chemero’s model of affordances, objectivity and subjectivity do not have separate ontological status; they co-exist and co-emerge relationally.

Building on Chemero (2003, 2009) and Rietveld and Kiverstein (2014) define an affordance as a relation between a feature or aspect of organisms’ material environment and ability available in their form of life. ‘Form of life’ is a notion adapted from the later Wittgenstein (1953). A form of life is a set of behavioral patterns, relatively robust on socio-cultural or biographical time scales, which is characteristic of a group or population. We might say that each species (or subspecies), adapted as it is to a particular niche and endowed with specific adapted abilities, constitutes a unique form of life. Different human communities, societies, and cultures, with sometimes strikingly different styles of engagement with the material and social world, constitute different forms of life. There are thus at least two ways to change the affordances available to an organism: (i) by changing the material aspects of its environment (which may vary from small everyday changes in its architecture or configuration to thoroughgoing niche construction) and (ii) by altering its form of life or allowing it to learn new abilities already available in that form of life (interacting in new ways with an existing niche by acquiring new abilities through various forms of learning).

Following recent theorizing on affordances (Rietveld, 2008a,c; Bruineberg and Rietveld, 2014; Rietveld and Kiverstein, 2014), we consider the distinction between the ‘landscape’ of affordances and the ‘field’ of relevant affordances. The claim is that, typically, organisms do not engage with one single affordance at a given time. The world we inhabit is instead disclosed as a matrix of differentially salient affordances with their own structure or configuration. The organism encounters the world that it inhabits as an ensemble of affordances, with which it dynamically copes and which it evaluates, often implicitly and automatically, for relevance. For an affordance to have ‘relevance’ here means that the affordance in question ‘solicits’ the individual, concrete organism by beckoning certain forms of perceptual-emotional appraisal and readiness to act. This occurs because affordances are both descriptive *and* prescriptive: *descriptive* because they constitute the privileged mode for the perceptual disclosure of aspects of the environment; and *prescriptive* because they specify the kinds of action and perception that are available, situationally appropriate and, in the case of social niches, expected by others.

The ‘landscape’ of affordances is the total ensemble of available affordances for a population in a given environment. This landscape corresponds to what evolutionary theorists in biology and anthropology call a ‘niche’ (Odling-Smee et al., 2003; Sterelny, 2007, 2015; Wilson and Clark, 2009; Fuentes, 2014). A niche is a position in an ecosystem that affords an organism the resources it needs to survive. At the same time, the niche plays a role vis-à-vis other organisms and their niches in constituting the ecosystem as a whole. A typical ecosystem (that is, a physical environment where organisms can live) has multiple niches, which have some degree internal structure: affordances have a variety of dynamics relationships (one thing leads to another, depends on, reveals, hides, enables, other possibilities for action; Pezzulo and Cisek, 2016). Thus, the niche is the entire set of affordances that are available, in a given environment at a given time, to organisms that take part in a given form of life. More narrowly, a niche comprises the affordances available to the group of organisms that occupy a particular place in the ecosystem—or, in the case of humans, the social world—associated with (and partly constituted by) a form of life.

The ‘field’ of affordances, on the other hand, relates to the dynamic coping and intelligent adaptivity of autonomous, individual organisms. The field refers to those affordances that actually engage the individual organism at a given time. Of those affordances available in the landscape, some take on special relevance as a function of the interests, concerns, and states of the organism. These relevant affordances constitute the field of affordances for each organism. They are experienced as ‘solicitations,’ in that they solicit (further) affective appraisal and thereby prompt patterns of ‘action readiness,’ that is, act as perceptual and affective prompts for the organism to act on the affordance (Frijda, 1986, 2007; De Haan et al., 2013; Rietveld et al., 2013). This engagement will vary in complexity, conformity, and creativity from pre-specified or pre-patterned ways of acting to “free” improvisation, as we shall see below^2^.

The field of affordances changes through cycles of perception and action. Changes in the situation that the organism engages give rise dynamically to different solicitations, as a function of the state of the organism, much the way a physical gauge field gives rise to different potentials as a function of the local forces (Sengupta et al., 2016). Consider the action of drinking a cup of coffee. The filled cup affords a gradient (grasping, sipping), that is, a potential for coupled engagement. When generated by the organism-environment system, this gradient can be experienced by the organism as a solicitation. The gradient is dissipated through engagement. The experience of satiation that follows drinking, combined with the fact that cup has been emptied, alter the field of affordances, which as indicated changes as a function of the states of organism and niche. Thus, the gradient is ‘consumed’ or dissipates after successful engagement.

### Meaning and Affordances

Not all affordances are of the same kind. Here we draw on Grice’s theory of meaning to suggest an approach to the varieties of cultural affordances in terms of their dependence on *content-involving conventions*. We argue that the affordances in human niches (what we call generally ‘cultural’ affordances) are of two distinct kinds: ‘natural’ and ‘conventional’ affordances.

Grice’s theory of meaning, elaborated in a series of papers in the philosophy of mind (Grice, 1957, 1969, 1971, 1989), and later refined by Sperber and Wilson (1986), Levinson (2000), and Tomasello (2014), is often termed ‘intention-based semantics’, or ‘implicature.’ On a Grician account, meaning lies in a speaker’s communicative intent; that is, in what she intends to convey through an utterance. Grice elaborated the first formula of his theory of meaning in these terms (using the subscript _NN_ to signify to ‘non-natural’):

“A meant_NN_ something by X” is roughly equivalent to “A uttered X with the intention of inducing a belief by means of the recognition of this intention” (Grice, 1989, p. 19)

Taking this model beyond the dyadic sphere of conversational implicature, Grice later attempted to explain how “timeless” (that is to say, durable and widely shared) conventions of meaning are recognized in a shared cultural repertoire:

“x means_NN_ (timeless) that so-and-so” might at a first shot be equated with some statement or disjunction of statements about what “people” (vague) intend (with qualifications about “recognition”) to effect by x (Grice, 1989, p. 220)

In the subsequent ‘relevance’ account, Sperber and Wilson (1986) translated this automatic ‘first shot’ recognition of conventional meaning as one in which human minds scan for salient, meaning-generating cues in the environment, and stop processing when the cues are secured.

**FIGURE 1:**
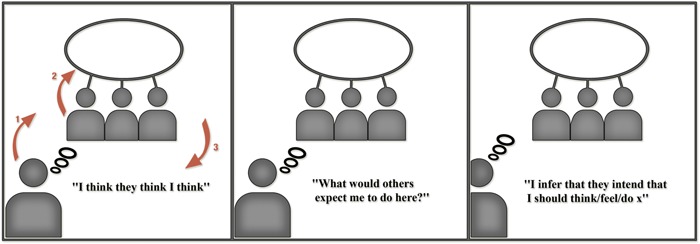
**Basic cognitive formula: three orders of automatic intentionality**.

**FIGURE 2:**
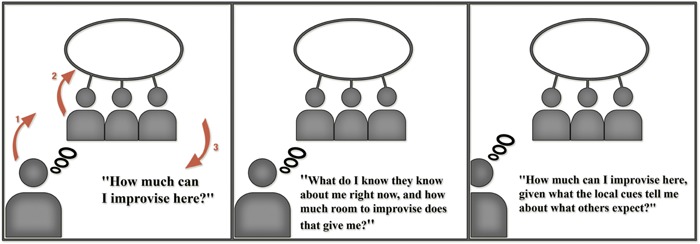
**Full cognitive formula: three orders of intentionality governing improvisational variations in action**.

Our model draws on Grice to describe the stabilization of cultural cues as affordances. Key to our approach is the implied ontological and epistemic status of other minds (that is, the intentions of ‘persons’) in the embodied cognitive work required in the ‘recognition,’ or more precisely, the enactment of meaning. Our proposal, then, is to follow Grice in understanding the thought, affect, and behavior of human agents as determined by implicit expectations about others’ expectations. Specifically, we argue that *humans behave according to the way they expect others to expect them to behave* in a given situation (see **Figure 1**)^3^. As we shall explicate below, we contend that humans operate (often pre-reflectively) within the landscape and field of possibilities for *variations* in action^4^ as a function of their expectations about what others expect of them in specific contexts (see **Figure 2**).

The importance of these revisions to Grice’s model of meaning to our framework for cultural affordances is to highlight the dependence of certain kinds of affordances on joint intentionality, and effective social and cultural normativity and conventionality, or equivalently, the shared expectations (both implicit and explicit) that codetermine the affordance landscape and local field dynamics. Grice (1957) distinguished between natural and non-natural forms of meaning, emphasizing the latter in most of his work. Natural meaning is a relation between two things that are correlated. Smoke ‘means’ fire because tokens of smoke reliably correlate with tokens of fire. Similarly, (certain kinds of) spots mean measles (understood not as the popular category but as the biomedically recognized infection with a particular virus). Non-natural meaning instead depends on the capacity of individual agents to exploit explicit and implicit social ‘conventions’ (in the wide sense of locally shared norms, values and moral frames, expectations, ontologies, etc.) to infer the intentional states of other agents and thereby engage them or engage aspects of the environment with them. Red traffic lights, in virtue of convention (and law), ‘mean’ stop, and hence afford (and mandate) stopping—and this is made possible by the specifically human mastery of recursive inferences, both explicit and implicit, that agents make about other agents (Tomasello, 2014).

Recent work on information processing has extended Grice’s framework to account for different kinds of *information* (Scarantino and Piccinini, 2010; Piccinini and Scarantino, 2011; Piccinini, 2015). A token informational vehicle *x* of kind *X* (that is, a sign, a pattern of neural activation, or what have you) carries ‘natural information’ about some information source *y* of kind *Y* just in case there are reliable correlations between *X* and *Y*. Natural information, in other words, cannot misrepresent, for it is non-semantic; it is not the kind of thing that can be simply true or false. Such information can be exploited and leveraged by a cognitive system to guide intelligent behavior. Conversely, ‘non-natural information’ (or as we prefer to put it, ‘conventional information’), pertains to semantic, content-involving representations that depend on social norms and cultural background knowledge. Non-natural information allows an agent to make a correct inference about some aspect of an intentional system, e.g., other agents, language and other symbolic systems such as mathematics, etc. Non-natural information is semantic in that it obtains in virtue of satisfaction conditions (e.g., truth conditions). A vehicle carries this kind of information about some state of affairs just in case some (explicit or implicit) shared convention, in the sense outlined above, links a vehicle to what it represents.

In the psychological and anthropological literature, affordances are usually understood as interactional properties between organisms and their environment that can be individually discovered in ontogeny without social learning. Chimpanzees, for example, rediscover how to crack nuts with rocks in each generation without vertical social transmission of skills (Ingold, 2000, 2001; Howes, 2011; Moore, 2013). Most of what humans do, in contrast, is learned socially and requires complex forms of coordination. We suggest, however, that successfully learned human conventions that govern action are also best conceptualized as affordances. Such affordances depend on *shared sets of expectations*, reflected in the ability to engage immersively in patterned cultural practices, which reference, depend on, or enact folk ontologies, moralities and epistemologies. We might call these ‘*conventional’ affordances*.

An empty street affords being walked on or driven on to the lone pedestrian or driver. Yet affordances, especially those depending on conventions, might differ depending on context. A red traffic light, as we have seen, affords an agent stopping, particularly in the presence of others, and especially in the presence (real or imagined) of police who are expecting to intervene. But a driver might alter her behavior as a result of not being seen by others. A red traffic light in an empty street at 4:00 AM, thus, might afford transgression of the stopping rule following an inference about the absence of other minds likely to judge the agent. Departing from Grice and earlier theories of information processing (Dretske, 1995), one might understand the notion of information as probabilistic: to carry information implies only the truth of a probabilistic claim (Scarantino and Piccinini, 2010; Scarantino, 2015). Although this account was developed for natural information, we extend it here to conventional information, given the prominence of social improvisation. ‘Conventions’ need not be explicitly formulated as rules, and may instead originate in the actors’ engagement with local backgrounds over time that is, from non-contentful developmental experiences, learning, or participation in social and cultural practices (Piccinini, 2015; Satne, 2015).

A cultural artifact may have multiple affordances according to its embedding in larger webs of relationships that are part of the individual’s history of learning and the expectations for the potential participation of others. Indeed, to operate with conventional affordances, agents must have shared sets of expectations—we must know what others *expect us to expect.*

Simple rule-governed models of sociality go on the assumption that conventions lead to stable, binary affordances, where satisfaction conditions are either met or not. However, cultural symbols and signs are usually polysemous and their interpretation depends on context. Moreover, variations in the way agents engage with affordances in practice, often license what we could term ‘skilled improvisation.’ Rules and conventions can be followed slavishly, selectively ignored, deliberately transgressed, or re-interpreted to afford new possibilities. Natural dispositions for shared intentionality in what Searle (1991, 1992, 1995, 2010) calls the deep background, on this view, give rise to cooperative action not only through convention but also through iterative variations governed by modes of engagement with cultural affordances (Terrone and Tagliafico, 2014).

## The Neurodynamics of Affordances

Some aspects of culture clearly involve content in the improvisational sense of the term: namely, those affordances that depend on conventions, social normativity, and the ability to improvise from a joint-intentional background enriched by cultural learning. Here, we aim to contribute to the effort to explicate the mechanisms by which basic minds are scaffolded into more elaborate content-involving processes. To explain agents’ engagement with contentful affordances requires a theory of cultural content and representations.

Our hypothesis, to be explicated below, is that feedback loops mediating shared attention and shared intentionality are the principal mechanism whereby cultural (especially conventional) affordances are acquired. Before proceeding, however, we must face an objection stemming from tensions between our enactivist-embodied-ecological framework and our aim of providing a theory for the acquisition of semantic content. We have suggested that conventional affordances depend on shared expectations, perspective-taking, and even mindreading abilities. However, proponents of radical embodiment and enactivism argue that cognition can be understood as the coupling of an organism to its niche through dynamical processes, without any need to invoke representational processes and resources like explicit expectations and mindreading (Varela et al., 1991; Gallagher, 2001, 2008; Thompson, 2007; Chemero, 2009). On these accounts, classical theories of cognition (Fodor, 1975; Marr, 1982), which modeled cognition as the rule-governed manipulation of internal representations, radically misconstrue the nature of agents’ intentional engagement with their worlds. The claim, then, is that much cognition can (indeed, must) be explained by appealing only to dynamical coupling between organism and environment.

Rejecting the claim that cognition necessarily involves representations, radical enactivists insist that basic cognitive processes (‘basic minds’) can function entirely without content (Thompson, 2007; Hutto and Myin, 2013). The argument, then, is that minds, especially basic minds like those of simple organisms (and many of the unreflective embodied engagements of more complex minds), do not require content. They only require adequate forms of coupling, which need bear no content at all. Adequate coupling only requires an organism to leverage correlations that are reliable enough to be exploited for survival. This poses a challenge to a theory like ours, which aims to explicate the acquisition of cultural content in the form of conventional affordances. In this section, we accommodate this radical minimalism about representations and semantic content while sketching a neural computational account of the scaffolding of cultural affordances.

### Computation, Representation, and Minimal Neural Models

Recent work on computation and neurodynamics helps to clarify the scope of radical arguments against content-involving, representational theories of cognition. Although older semantic theories view computation as the processing of representations (with propositional content and satisfaction conditions) more recent theories do not make this assumption. The ‘modeling view’ of computation (Grush, 2001; Shagrir, 2006, 2010; Chirimuuta, 2014) suggests that computation in physical systems (calculators, digital and analog computers, neural networks) employs a special kind of minimal, structural or analogical model based on statistical correlations (O’Brien and Opie, 2004, 2009, 2015). On this view, a computational process is one that dynamically generates and uses a statistical model of a target domain (say, things in the visual field). The model is said to ‘represent’ that domain only in the sense that the relations between its computational vehicles (digits, neural activation patterns, or what have you) preserve the higher-order statistical, structural-relational properties of the target domain, which can be leveraged to guide adaptive action. We might call this ‘weak’ (non-propositional) content, based on structural analogy between vehicle and target domain (O’Brien and Opie, 2004, 2009, 2015). Such statistical models are much more minimalistic than traditional representational theories of mind, which require that internal representations bear propositional content (Fodor, 1975). Even more minimalistic accounts of computation are available. Computation can be defined mechanistically, as the rule-governed manipulation of computational (rather than representational) vehicles (Milkowski, 2013; Piccinini, 2015). On the mechanistic account, computations (digital, analog, neural) can occur without *any* form of semantic content (Scarantino and Piccinini, 2010; Piccinini and Scarantino, 2011).

Thus, some of the newest theories of computation are minimalistic about the representational nature of neural processes. Whether the modeling-structural and the mechanistic minimal statistical models deserve the label ‘representation’ is debatable (Anderson and Chemero, 2013; Piccinini and Shagrir, 2014; Hutto, 2015; Clark, 2016). To some degree the conflict may be merely terminological. What matters for our purposes is to note that the minimalistic statistical-computational models in the cognitive system can be leveraged to guide skilled intelligent, context-sensitive, adaptive behavior. This provides additional weight to the claim that basic minds are without strong, propositional, semantic content (Hutto and Myin, 2013; Hutto et al., 2014).

While this may be the case, human societies clearly transact in content-laden representations. We use language replete with images, metaphors and other symbols to tell stories and narrate our lives. We imagine particular scenarios or events, and we think about, describe, elaborate and manipulate these images or models in ways that treat them as pictures or representations of possible realities. Importantly, even on the radical view on offer here, nothing *precludes* such content-involving cognition. In recent discussions around the natural origins of content, it is hypothesized that neural computations can come to acquire representational content when coupled adequately to a niche or milieu through dense histories of causal coupling (Hutto and Myin, 2013; Hutto and Satne, 2015; Kirmayer and Ramstead, 2016). We suggest that immersive involvement of agents in patterned cultural practices during development, and the subsequent practice of the abilities acquired in enculturation, allows for the acquisition of stable cultural affordances. In the case of human beings, whose learning is mostly social, the function of the neural computations performed by a system becomes that of interfacing adequately with both representational and non-representational aspects of culture so as to guide appropriate behavior.

### Free-Energy and the Neurodynamics of Affordances

The framework we think can account for the acquisition of cultural affordances by agents rests on recent work in computational neuroscience and theoretical biology on the ‘free-energy principle.’ The free-energy principle is a mathematical formulation of the tendency of autonomous living systems to adaptively resist entropic disintegration (Friston et al., 2006; Friston, 2010, 2012a, 2013a,b; Sengupta et al., 2016). This disintegration can be thought of as the natural tendency of all organized systems (which are by their nature far-from-equilibrium systems) to dissipate, that is, to return to a state of low organization and high entropy or disorder—in other words, to return to (thermodynamic) equilibrium. The free-energy principle states that the dynamics of living organisms are organized to maintain their existence by minimizing the information-theoretic quantity ‘variational free-energy.’ By minimizing free-energy, the organism resists entropic dissipation and maintains itself in its phenotypical steady-state, far from thermodynamic equilibrium (death).

**FIGURE 3:**
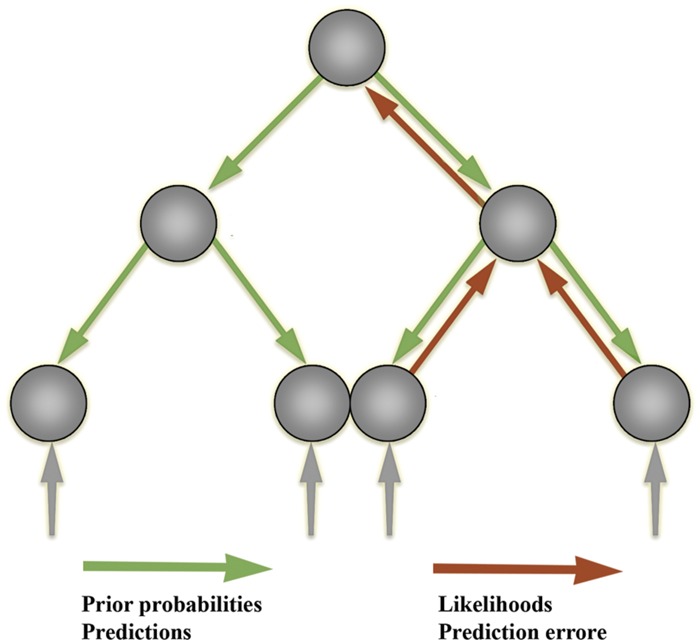
**Hierarchical prediction error minimization frameworks.** In the predictive processing approach, the main activity of the nervous system is to predict upcoming sensory states and minimize the discrepancy between prediction and sensory states (‘prediction errors’). The information propagated upward to higher levels for further processing consists only in these prediction errors.

**FIGURE 4:**
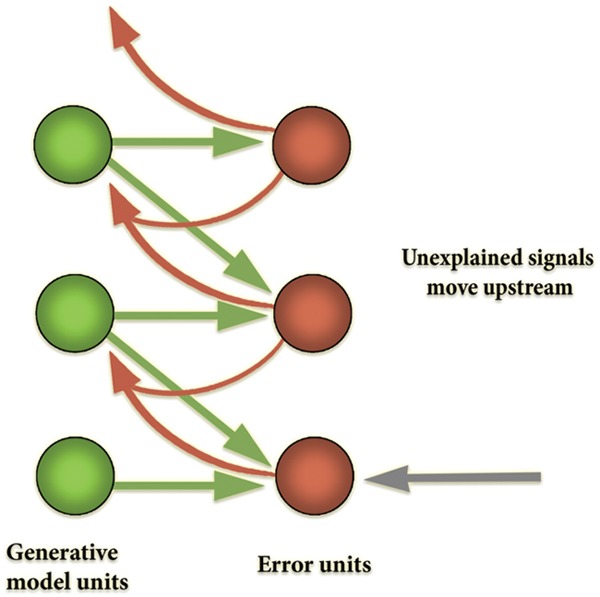
**A diagram of Bayesian inference in predictive processing architectures.** The dynamics of such systems conform to the principles of the Bayesian statistical inference framework. The Bayesian statistical framework is central to predictive processing architectures, for the latter assume that neural network interactions operate in a way that maximizes Bayesian model evidence. Bayesian methods allow one to calculate the probability of an event taking place by combining the ‘prior probability’ of this event (the probability that such an event takes place before considering any evidence) with the ‘likelihood’ of that event, that is, the probability of that event given some evidence. This allows the Bayesian system to calculate the ‘posterior probability’ of the event, that is, the revised probability given any new available evidence. Prior probabilities are carried by predictions (green arrows) issued by the generative model units (green units). Likelihoods are carried by prediction errors (red arrows) issued by the error units (red units). In the ‘empirical Bayes’ framework, the system can then use the posterior obtained from one iteration as the prior in the next iteration. Predictions issued from the generative models, which encode prior beliefs, propagate up, down, and across the hierarchy (through backwards and lateral connections) and are leveraged to guide intelligent adaptive action-perception. This leveraging is achieved by canceling out (or ‘explaining away’) discrepancies, which encode likelihood, through rolling cycles of action-perception. This same process allows the system to learn through plastic synaptic connections, which are continuously updated through free-energy minimization in action-perception. The system thus continuously and autonomously updates its ‘expectations’ (Bayesian prior beliefs) in rolling cycles of action-perception.

One application of the free-energy principle in computational neuroscience is a family of models collectively referred to as ‘hierarchical predictive processing’ models, which instantiate a more general view of the brain as a ‘prediction machine’ (Frith, 2007; Friston and Kiebel, 2009; Friston, 2010, 2011, 2012b; Bar, 2011; Hohwy, 2013; Clark, 2016; for empirical evidence, see Adams et al., 2016). In this framework, the brain is modeled as a complex dynamical system, the main function of which is to ‘infer’ (in a qualified sense) the distal causes of its sensory stimulation, starting only from its own sensory channels. The strategy employed by the brain, according to this view, is to use a ‘generative model’ of the distal causes and engage in self-prediction (Friston, 2010; Eliasmith, 2005). That is, the system’s function is to predict the upcoming sensory state and compare it the actual sensory state, while minimizing the difference between these two distributions (predictions and prediction errors) through ongoing modification of predictions or action on the environment (see **Figures 3** and **4**).

‘Generative models’ are minimal statistical models, of the kind discussed above. The use by a system of generative models need not entail semantic content. Their function is to dynamically extract and encode information about the distal environment as sets of probability distributions. The information involved here can be natural or conventional in kind. The only entailment is that the system or organism must leverage its generative model to guide skilled intentional coupling. The system uses this generative model to guide adaptive and intelligent behavior by ‘inverting’ that model through Bayesian forms of (computational, subpersonal) inference, allowing it to leverage the probability distributions encoded in the model to determine the most probable distal causes of that distribution and to act in the most contextually appropriate way (Friston, 2010; Hohwy, 2013; Clark, 2016).

**FIGURE 5:**
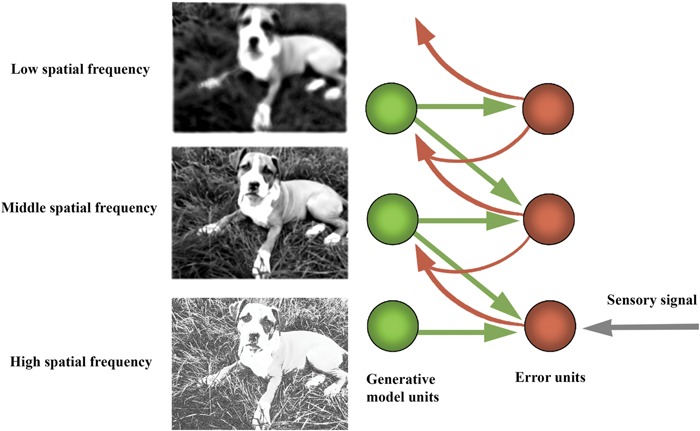
**Diagram of hierarchical structure of the predictive processing networks.** Predictive networks have hierarchical structure in the sense that their processing is layered. The layered (hierarchical) structure of the generative model allows the model to capture the nested structure of statistical regularities in the world. This inferential architecture effectively allows the system to leverage new information dynamically and implement a ‘bootstrapping’ process, whereby the system extracts its own priors from its dynamic interactions with the environment. Computationally, each individual layer has the function of extracting and processing information leveraged to cope with regularities at a given level or scale. In this example, information about the visual scene is decomposed into high, medium, and low spatial frequency bands. Typically, low spatial frequency features change at a faster than high spatial frequency features. As such, lower spatial frequency information is encoded higher up in the processing hierarchy, to guide lower-level, faster processing of higher spatial frequency information. The hierarchical or layered statistical structure of the generative model enables it to recapitulate the salient statistical structure of those systems to which it is coupled. As discussed in the text, this need not imply semantic content (but does not exclude it either).

How does this inversion take place? Generative models are used to generate a prediction about the upcoming sensory distribution. Between the predicted and actual sensory distributions, there almost always will be a discrepancy (‘prediction error’), which ‘tracks’ surprisal (in the sense that, mathematically, it is an upper bound on that quantity). The free-energy principle states that all living systems act to reduce prediction error (and thereby implicitly resist the entropic tendency toward thermodynamic equilibrium—dissipation and death). This can occur in one of two complementary ways: (i) through action, where the best action most efficiently minimizes free-energy by making the world more like the prediction (‘active inference’); and (ii) through perception and learning, by selecting the ‘hypothesis’ (or prediction, which corresponds to the probable distal cause of sensory distribution) that most minimizes error, or changing the hypotheses when none fits or when one fits better (Friston, 2011, 2013a; Friston et al., 2012a,b; Friston and Frith, 2015a,b). Given that generative models embody fine-grained statistical information about the distal environment at different scales, the top-down prediction signals (produced by higher levels in the processing system) provide crucial contextualizing information for the activity of lower levels in the predictive hierarchy, rendering the feedforward error signal contextually sensitive and adaptive (see **Figure 5**).

The representational minimalism of embodied generative models nicely complements the representation-sparse phenomenology of affordances. Such minimal models might be described as exploiting (non-semantic) information *for* affordances, rather than (semantic) information *about* affordances (van Dijk et al., 2015); that is, the sensory array only carries information given certain uses of it by organisms (i.e., being a statistical proxy). The ‘internal representations’ involved here might best be thought of as transiently ‘soft-assembled neural ensembles,’ adequately coupled to environmental affordances (Anderson, 2014).

It can be argued that predictive processing models complement enactivist and radical embodied approaches and are compatible with minimalism about representations, provided we do not interpret the statistical computations and error signal processes in a strong semantic, content-involving sense (Hutto and Satne, 2015; Kirchhoff, 2015a,b, 2016; Kirmayer and Ramstead, 2016). Generative models are simply *embodied statistical models* that are dynamically leveraged to guide intelligent adaptive behavior.

Generative models are embodied at different systemic levels and timescales, in different ways. As indicated, at the level of the brain, the predictive hierarchical architecture of neural networks come to encode statistical regularities about the niche, which allow the organism to engage with the field of affordances in adaptive cycles of action-perception. But the embodiment of generative models does not stop at the brain. Indeed, one radical implication of the free-energy principle is that the *organism itself is* a statistical model of its niche (Friston, 2011, 2013b). States of the organism (i.e., its phenotype, behavioral patterns, and so forth) come to statistically model the niche that it inhabits over evolutionary timescales (Badcock, 2012). Thus, phylogeny conforms to the free-energy principle as well, because the effect of natural selection is to select against organisms that are poor models of their environments. Those organisms that survive and thrive are those that embody, in this literal sense, the best generative models of their niche. Organism phenotypes can be described as conforming to the free-energy principle over developmental timescales in morphogenesis as well (Friston et al., 2015b). Generative models are thus not only ‘embrained,’ but embodied in an even stronger sense, over the timescales of phylogeny and ontogeny. This strong embodiment allows one to interpret free-energy approaches in a non-internalist way and to counter some objections raised against earlier formulations of predicting processing approaches (e.g., Hohwy, 2013; Clark, 2016). This multilevel embodiment of the generative model, as we shall argue below, extends to the concrete, material, human-designed milieus (or ‘designer environments’) in which humans operate.

Some generative models (in this wide sense) involve semantic content and others do not (they involve something more minimal than satisfaction conditions, i.e., reliable covariation). The study of minds without content is compatible with more extensively content involving forms of (social and cultural) cognition that are scaffolded on such basic minds through processes of social learning and enculturation.

On the radical enactivist account, content-involving forms of intentionality emerge in the context of certain cultural practices in human forms of life (Hutto and Satne, 2015). Many of these practices involve multi-agent situations in which proper engagement requires forms of implicit perspective-taking and perspective-sharing (Sterelny, 2015). In some cases, such practices can involve explicit ‘mindreading’ as well, that is, inferring the beliefs, intentions, and desires of other agents *as such* (Michael et al., 2014). There is a long-running debate among anthropologists over the extent to which inferences about other people’s mental states (as opposed to, say, bodily states) may reflect a folk psychology that is more pronounced among modern Western peoples (Robbins and Rumsey, 2008; Rumsey, 2013). This ‘transparency of mind’ folk psychology is contrasted in the literature with so-called ‘opacity doctrines’ found in other cultures, in which people’s interior states are said to be ‘opaque,’ or unknowable. As recent multi-systems account of social cognition have shown, however, situations involving novel cues or too many orders of intentionality will often trigger ‘higher’ cognitive resources and compel humans to think about other people’s intentions as such (Michael et al., 2014). Engagement with affordances in the human niche also often requires ‘mindshaping,’ as our interpretation of other agents’ intentional profiles in turn shapes those same profiles through interpersonal loops (Sterelny, 2007, 2015; Zawidzki, 2013). Perspective-taking can be implicit and embodied in that organisms can act on situations by leveraging minimal models that encode information about other agents and their behavior without entailing the presence of semantic content (i.e., having satisfaction conditions). But this is not incompatible with the claim that perspective-taking and mindshaping abilities, in the human niche, often involve symbolically and linguistically mediated forms of communication, which substantially change the kind of affordance landscape available to human agents (Kiverstein and Rietveld, 2013, 2015).

Although the perspectivist focus on the dynamic embodied enactment of meaning in a shared social world is central to our understanding of cultural affordances (Gallagher, 2001, 2008; Fuchs and De Jaegher, 2009), our contention is that the acquisition of representational content in ‘epidemics’ of socially shared representations (Sperber, 1996; Claidière et al., 2014) entails that cognitive agents must be endowed with a neural-computational scaffolding adequate to such activities^5^. Even though basic cognition (and indeed, some forms of ‘higher’ cognition; Hutto and Myin, 2013) may be without content, given the symbolic and linguistic nature of human experience and culture, the human cognitive system must be equipped with the neural-computational resources needed to adequately couple with shared social representations, if we are to account for how the latter are transmitted stably and reliably. Semantic content is acquired through dense histories of embodied engagement with the environment. For humans, this involves participation in patterned, linguistically and symbolically mediated practices—which include patterns of shared attention and shared intentionality.

### Predictive Processing and Attention

One aspect of the architecture of predictive processing is crucial for our account of cultural affordances: the predictive processing model specifies a deep functional role for *attention*. Attention, on the predictive processing account, is modeled as ‘precision-weighting,’ that is, the selective sampling of high precision sensory data, i.e., prediction error with a high signal-to-noise ratio (Feldman and Friston, 2010). The efforts of the cognitive system to minimize free-energy operate not only on first-order, correlational statistical information about the distal environment, but on second-order statistical information about the signal-to-noise ratio or ‘precision’ (that is, inverse variance) of the prediction error signal as well. This allows the system to give greater weight to less noisy signals that may provide more reliable information. Based on this information, the cognitive system balances the gain (or ‘volume’) on the units carrying prediction errors at specific levels of the hierarchy, as a function of precision. This control function, in effect, controls the influence of encoded prior beliefs on action-perception (Friston, 2010). Greater precision means less uncertainty; the system thus ‘ups the volume’ on high precision error signals to leverage that information to guide behavior. Attention, then, is the process whereby synaptic gain is optimized to ‘represent’ (in the sense of reliably co-varying with) the precision of prediction error in hierarchical inference (Feldman and Friston, 2010; Clark, 2016).

Precision-weighting is centrally important in these architectures and has been proposed as a mechanism of *neural gating*. Gating is the process whereby effective connectivity in the brain (Friston, 1995, 2011), that is, the causal influence of some neural units on others, is controlled by the functioning of distinct control units (Daw et al., 2005; Stephan et al., 2008; den Ouden et al., 2010). These are called ‘neural control structures’ by Clark (1998) (For assessments of the empirical evidence, see: Kok et al., 2012, 2013; Friston et al., 2015a). Attention-modulated ‘gating’ is the central mechanism that allows for the formation of transient task- and context-dependent coalitions or ensembles of neural units and networks (Sporns, 2010; Park and Friston, 2013; Anderson, 2014).

**FIGURE 6:**
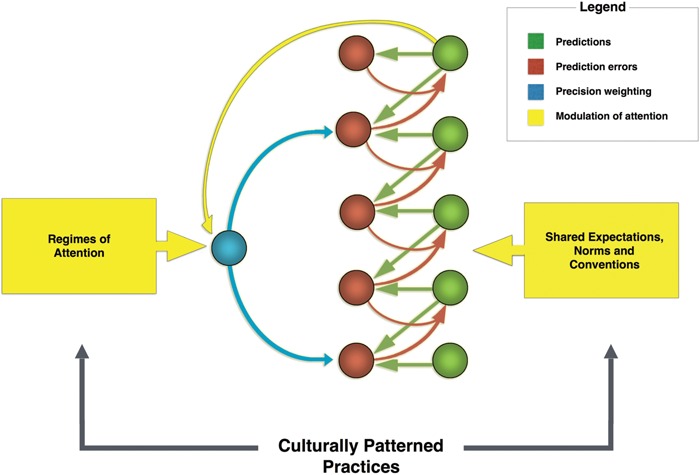
**A diagram of the looping effects that mediate cultural affordance learning.** Regimes of attention, a central kind of patterned cultural practice, and higher level expectations encoded in higher levels of the cortical hierarchy, guide agents’ attentional styles. In the free-energy framework, attention is modeled as precision-weighting and has the function of controlling activation across the various levels of the cortical hierarchy by tuning the gain on error units (that is, they realize the function of gating effective connectivity in the brain). In turn, differences in how attention is deployed (through gating) lead to varying salience landscapes and to different expectations being encoded in the predictive hierarchy. Based in part on Figure 1 in Friston et al. (2014).

Thus, in the predictive processing framework, attention is the main driver of action-perception. Clark (2016, p. 148ff) describes possible implementations of this scheme in the brain. Much like for first-order expectations, the system encodes expectations about precision in the generative model, presumably in the higher levels of the cortical hierarchy (Friston et al., 2014). These signals, which carry context-sensitive second-order statistical information, then guide the balancing act between top–down prediction signals from the generative models and bottom–up error signals in attention (see **Figure 6**).

It has been argued that predictive processing models offer a plausible implementation for the neural-computational realization of affordance-responsiveness in the nervous system (Clark, 2016). As we shall see below, the free-energy model provides a mechanistic implementation of the dynamical gradient generation and consumption conception of affordance engagement examined above (Bruineberg and Rietveld, 2014). Free-energy is minimized through action and perception by the predictive processing hierarchy, which provides a mechanistic implementation of the descriptive-prescriptive aspect of affordances.

## Cultural Affordances and Shared Expectations

We lack comprehensive accounts of how the conventions that give rise to sociocultural affordances are successfully internalized, both as implicit knowing how and explicit knowing that. As Searle and others (Sterelny, 2007; Tuomela, 2007; Tomasello, 2014; but see Zahavi and Satne, 2015) have shown, and as our models suggests, it takes higher-order levels of intentionality, meta-communication, and perspective-taking in order for symbolic conventions to be used and manipulated—and for more complicated, self-referential thinking (“I know that she thinks that I believe that she intends to *X*,” etc.), collective intentionality, and multiple orders of mindreading.

The question for the present essay is how this framework can be scaled up to account for cultural and social cognition and learning. The everyday phenomenology of affordances is one of possibilities for action and their variations; in other words, of *expecting* certain nested action possibilities and prescriptions for action. In effect, the phenomenology of affordances is a phenomenology of *expectations* about available and appropriate agent-environment couplings. The neural-computational models derived from the free-energy principle traffics in predictions and conditional probability distributions (called ‘beliefs’ in Bayesian probability theory, without any claim to correspond to the folk psychological notion). Arguably, the phenomenological correlate of these Bayesian beliefs can, at least at some (presumably higher) levels of the predictive hierarchy, be thought of as (or at least codetermine) *agent-level expectations*. Our remarks below focus on clarifying how the social scaffolding of agents leads to their acquisition of representational content in regimes of shared attention.

### Skilled Intentionality and Affordance Competition

On the radical embodied view, the central feature of the dynamic relations between organisms and environment is the tendency of the organism to move toward an ‘optimal grip’ on the situation. The optima in question, as nearly everywhere in biology, are local optima, rather than a single global optimum. Under the free-energy framework, the ‘optimal grip’ can be understood as the pattern of action-perception that most minimizes variational free-energy. The free-energy minimizing dynamics of the predictive hierarchy might be described as a kind of weighted or biased competition between different affordances, the ‘affordance competition’ hypothesis (Cisek, 2007; Cisek and Kalaska, 2010; Pezzulo and Cisek, 2016). This model of action selection theorizes that the cognitive system appraises different trajectories for motor action simultaneously during action selection (that is, appraising a whole field of affordances in parallel and dynamically settling on the most salient affordance).

Sport science provides an illustration of this tendency toward optimal grip (Hristovski et al., 2006, 2009; Chow et al., 2011). Studies of the dynamic interplay between a boxer’s stance and position, and the action possibilities available to them as a function of stance and position, have shown that punching bags afford different kinds of strikes to boxers as a function of the distance between boxer and punching bag. Boxers tend to move their bodies to an optimal distance from the punching bag, specifically, one that affords the greatest variety of strikes. This is a case of moving toward optimal grip. When observing a painting, we also move our bodies and our gazes in a way that maximizes our grip on the scene or details observed. We might call such dynamic adaptive engagement with field of affordances in rolling cycles of action-perception ‘skilled intentionality’ (following Merleau-Ponty, 1945/2012; Rietveld, 2008b, 2012; Bruineberg and Rietveld, 2014).

Using the theoretical frameworks of dynamical systems and self-organization, Bruineberg and Rietveld (2014) have conceptualized this skilled intentionality as a kind of coping with the potentials that well up in the field of affordances, as a result of the dynamic relations between organism (with its phenotypical states, its states of action readiness, its concerns, etc.) and environment. More specifically, they suggest that skilled intentionality is the generation and reduction (or ‘consumption’) by the organism of a ‘gradient’ or potential tension in the field of affordances (which can be modeled using attractor dynamics). We sketched this approach in Sections “Landscapes and Fields” and “Meaning and Affordances,” without the free-energy framework. The full significance of dissipative dynamics in the field of affordances can now be appreciated.

Affordances that are relevant to the organism at a given time (solicitations) drive system dynamics by soliciting rolling loops of action-perception and are prescribed and consumed or dissipated by those very dynamics (Tschacher and Haken, 2007). That is, solicitations are equivalent to potentials in the field of affordances, which act as attractors on the organism-environment dynamics, changing those affordances to which the organism is selectively open and receptive. The solicitations with which the organism engages, on this view, is the one that most effectively minimizes free-energy. Affect, attention, and affordances interact to sculpt a field of solicitations out of the total landscape of available affordances, adaptively and dynamically moving the organism toward an optimal grip on situations through action-perception. As the organism moves along a gradient toward an optimal grip, the gradient dissipates. The field of affordances thus changes dynamically along with perception-action and changes to states of the organism and environment. Responsiveness to the field, informed by states of the organism and environment, prescribe modes of optimal coupling. The radical embodied conception of cognition as skilled intentionality, then, can be modeled using systems theoretical models as a kind of selective responsiveness to salient available affordances or solicitations, modulated by states of the organism (concerns, interests, abilities) and states of the environment. This framework effectively bridges the descriptive levels of phenomenology, system dynamics, and cognitive functions or mechanisms.

To date, most work on affordances has focused on motor control and basic behaviors related to dynamical embodied coping (e.g., Chemero, 2009; Cisek and Kalaska, 2010; Pezzulo and Cisek, 2016). For a theory of cultural affordances, the notion of affordances must be extended to more complex features of the social and cultural niche inhabited by humans (Heft, 2001; Bruineberg and Rietveld, 2014; Rietveld and Kiverstein, 2014). Quintessential human abilities like language, shared intentionality, and mind-reading/perspective-taking emerge from human forms of life and are patterned by human sociocultural practices (Roepstorff et al., 2010), which in turn involve sophisticated forms of social cognition. We live in a landscape of cultural affordances.

### Shared Expectations, Local Ontologies, and Cultural Affordances

The upshot of our discussion so far is a general concept of skilled intentionality as selective engagement with a field of affordances supported by embodied generative models. Skilled intentionality is a graded phenomenon. At one extreme, skilled intentionality consists in contentless direct coping. It has been suggested that this most basic form of intentionality, which Hutto and Satne (2015) call ‘ur-intentionality,’ acquires its tendencies for selective targeted engagement with the world in a ‘teleosemiotic’ process shaped by evolutionary history^6^. At this extreme, the only information (and affordances) needed are of the natural kind (exploitable reliable correlation). At the other extreme, we find stereotypical human intentionality, that is, symbolically dense and strongly content-involving forms of collectively and conventionally rooted intentionality (Kiverstein and Rietveld, 2015), which involves conventional information and affordances. This is a spectrum, and all points between these extremes are viable (at least prima facie). The teleological basis of this variation might be the needs, concerns, and abilities relevant to a given form of life, (Bruineberg and Rietveld, 2014; Rietveld and Kiverstein, 2014), in specific social niches with their own idiosyncratic shared representations, symbols, etc.

Our claim here is that cultural affordances (especially conventional ones) form a coordinated affordance landscape, which is enabled by sets of embodied expectations that are shared by a given community or culture. Social niches and cultural practices generally involve not isolated, individual affordances or expectations but local landscapes that give rise to and depend on shared expectations. We submit that these shared expectations—implemented in the predictive hierarchies, embodied in material culture, and enacted in patterned practices—contribute to the constitution of the landscape of affordances that characterizes a given community or culture. Indeed, shared expectations modulate the specific kinds of intentionality that are effective in a given community, determining the forms taken by skilled intentionality, especially the shared skilled intentionality of the kind that constitutes a patterned sociocultural practice.

Patterned practices are specific ways of doing joint activities in domain-specific material-discursive environments (Roepstorff et al., 2010). Echoing recent work on the natural origin of semantic content (Hutto and Satne, 2015; Sterelny, 2015), we hypothesize that such ontologies, as socially shared and embodied expectations, come to be acquired by the individual agent through their participative immersion in specific patterned practices available in multi-agent, symbolically and linguistically mediated forms of social life.

Building on work in cognitive science as well as by Hacking (1995, 1999, 2002, 2004), Kirmayer and colleagues have argued for an embodied, enactivist approach to the study of the multilevel feedback or ‘looping’ effects involved in jointly-mediated narratives, metaphors, forms of embodiment, and mechanisms of attention (Kirmayer, 2008, 2015; Seligman and Kirmayer, 2008; Kirmayer and Bhugra, 2009; Kirmayer and Gold, 2012). In human life, the regularities to which agents are sensitive are densely mediated (and often constituted) by cultural symbols, narratives, and metaphors, which may explicitly reference or tacitly assume particular ontologies. These mechanisms shape social experience and in turn are shaped by broader social contexts.

Elsewhere, we have suggested that local, culturally specific ontologies can be understood as sets of shared expectations (Kirmayer and Ramstead, 2016). A ‘local ontology’ can be defined as a mode of collective expectation: agents expect the sociocultural world to be disclosed in certain ways rather than others and to afford certain forms of action-perception and nested variations to the exclusion of others. A local ontology, then, is a set of expectations that are shared by members of a cultural community. We claim that these sets of shared expectations are installed in agents through patterned practices that result in enculturation and enskillment. In the framework explored above, these ontologies codetermine the exact affordances that are available in a given niche, for they prescribe specific ways of being, thinking, perceiving, and acting in context that are situationally appropriate.

These local ontologies need not be explicitly formulated as metaphysical theories. They are more often implicit and acquired through participation in patterned practices and the enactment of customs and rituals, or embodied in the social material reality itself (as symbols, places, stories). Such distinctively human practices take place in social niches rich with narratives, symbols, and customs, which enable individuals to respond cooperatively and, at times, to infer other agents’ states of mind. Such practices may underlie everyday processes of person-perception. For example, as noted in the introduction, by age 5, children have acquired local ontologies and categories of personhood—which reproduce the dominant set of biases, expectations, and representations of their cultures—showing preference for dominant group culture often without being explicitly taught to do so, and despite their caregivers not consciously holding such views, even when these biases are not consonant with their minority identities (Clark and Clark, 1939; Kinzler and Spelke, 2011). These tacit views of others may arise both from the ways in which local niches are structured by social norms and conventions and from regimes of attention and interpersonal interactions shaped by cultural practices (Richeson and Sommers, 2016). Biases in person-perception will, in turn, influence subsequent social interaction and cooperative niche construction in a cognitive-social loop (Sacheli et al., 2015).

As discussed above, a number of theorists of embodied cognition have criticized the view that intersubjective interactions require that human beings be endowed with the capacity for mind-reading, opting instead for an explanation in terms of embodied practices and coupling (Gallagher, 2001, 2008; Fuchs and De Jaegher, 2009). Although we readily grant the importance of such embodied coping for basic minds on which more elaborate cognition can be scaffolded, we advocate a middle ground that posits both embodied contentless abilities and more contentful mindreading abilities (Michael et al., 2014; Tomasello, 2014; Sterelny, 2015; Veissière, in review). Indeed, the framework we have proposed, which posits predictive processing hierarchies apt to engage with both natural and conventional information and affordances, can accommodate both modes of cognition. The view that human societies rely on explicit and implicit forms of mindreading does not commit us to intellectualism or to a strong content-involving view. The shared enactment of meaning, involving expectations about other agents, comes to constitute the shared, taken-for-granted meaning of local worlds, which in turn feeds back, in a kind of looping effect, to developmentally ground and scaffold the enactments of meaning by individual agents, by altering the shared expectations that are embodied and enacted in the social niche (Kirmayer, 2015). These shared ontologies shape experience by changing the abilities and styles of action-perception of encultured agents.

### Shared Expectations and Implicit Learning

We have already appealed to Grice’s theory of meaning to clarify some aspects of affordances. Affordances come in a spectrum, ranging from those that depend only on reliable correlation to those that depend on shared sets of expectations. Grice’s account, as improved by others (Sperber and Wilson, 1986; Levinson, 2000; Tomasello, 2014), can help account for how we successfully learn to detect and selectively respond to context in situations that involve higher order contextual appraisal, including perspective-taking and reading of other’s goal-directed intent and actions. In higher-order, rule-governed semiotic contexts, the actual presence of others is not necessary for inferences to be made about the ‘correctness’ of affordances in terms of their correspondence to others’ expectations, norms or conventions. The general internalized idea of how others would interpret a situation and context (or how a culturally competent actor would respond) suffices for ‘meaning’ to be derived or inferred.

Most of us have never been explicitly taught precisely how to behave, sit, move, speak, take turns, and interact with others in shared spaces such as metros, elevators, hallways, airplanes, university classrooms, bars, dance floors, janitors’ closets, or the many other spaces we know not to enter. As mentioned in the introduction to this essay, children acquire the dominant social norms and appropriate behavioral repertories and responses without explicit instruction. Although we do occasionally receive explicit instructions, these do not seem necessary for normal social functioning; as Varela (1999) pointed out, we have acquired the implicit ‘know how’ to act appropriately. That is, human beings acquire characteristic, stereotypical ways of doing and being in response to social contexts; in a sense, each of these constitutes habitual ‘micro-selves’ as we variously engage the world as our ‘getting-on-the-bus-self’ to our ‘having-lunch-self,’ etc., where each self is a style of situationally adequate and socially appropriate coupling to a context. How do we acquire the ability to selectively detect and respond to such sociocultural affordances? Or to rephrase the question in anthropological terms: how do us come to be socialized or enculturated for participation in shared worlds of expectations?

The highly stable conformity of behavior in all of these contexts goes beyond direct imitation (Michael et al., 2014). Many everyday situations involve coordinated action among many participants. Although some forms of coordinated group action can occur entirely through individual responses to local impersonal affordances (e.g., the swarming of birds), in order to read and master the social cues and scripts in complex human settings, the actors involved need to grasp the situation from the perspective of other actors. This perspective-taking is essential if each actor’s appraisal of the situation is to have any counterfactual depth with regard to explicit social norms (e.g., inferring that one’s behaving differently would fail to conform to others’ *expectations* about correct behavior). However, as argued above, in some instances this perspective-taking might not involve explicit, content-involving processes; the expectations might simply be encoded and leveraged for the generation of adaptive behavior without mentalistic assumptions being made about agents at an explicit, conscious level. Thus, in any case, for a given space to afford the same engagements to a given population, that community must come to share a set of collective expectations—indeed, shared expectations about *others’ expectations about our expectations*, and so forth.

## Regimes of Shared Attention and Shared Intentionality

The framework we have outlined for cultural affordances allows us to reconsider the natural origins of content. We hypothesize that the central mechanism whereby cultural affordances are acquired, especially conventional, content-involving affordances, consists in the looping or feedback relations between shared intentionality and shared attention. Shared intentionality is enacted in various concrete, materially embedded cultural practices and embodied as shared sets of expectation. Shared attention is one such form of shared intentionality. We suggest that shared attention is crucial because directed attention modulates the agent’s selective engagement with the field of affordances. Given the nature of the predictive hierarchy, to wit, to extract explicit and implicit statistical information, directing an agent’s attention is tantamount to determining which expectations (Bayesian prior beliefs) will be encoded in the hierarchy. This, in turn, leads to different sets of abilities being implemented by the gating mechanisms of the predictive hierarchy. Under the free-energy principle, action-perception is guided attention (precision-weighting), and the gating process that is realized by attention itself rests on the expectations encoded in the generative models embodied by the organism. These high-level expectations about precision, which modulate allocations of attention (and thereby determine action-perception through gating), are leveraged to guide skillful intentional behavior. The sets of expectations embodied and enacted by organisms change the field of affordances. This mechanism, we submit, is exploited by culture in the acquisition of cultural affordances.

### Gating, Abilities, and Affordances

In the framework outlined above, we followed Rietveld and Kiverstein (2014) in defining an affordance as a relation between a set of features or aspects of the organism’s material environment and the abilities available in that organism’s form of life. We are now in a position to better define ability in terms of a gating control pattern, that is, a sequenced or coordinated process. An ability is simply the capability of an organism to coordinate its action-perception loops to skillfully engage an affordance in a way that is optimal under the free-energy principle. An ability, then, in the free-energy framework, includes a pattern of attention, in the specific sense employed by the free-energy framework. We use the term ‘attention’ not in the folk-psychological sense, as that effort or mechanism that allows us to attend to specific aspects of experience, but as the mechanism of precision-weighting that mediates neural gating and allows the agent to engage with specific affordances in action-perception cycles. Attention, in our technical sense, therefore modulates effective connectivity and, as such, determines the trajectories taken by the rolling cycles of action-perception. Typically, in the case of human agents, such patterns of attention are acquired over development.

We conjecture that we acquire our distinctively human abilities from our dense histories of temporally coordinated social interaction and shared cultural practices (Tomasello et al., 2005; Roepstorff, 2013). Attentional processes are central to this enculturation and installation of shared semantic content. In particular, the landscape of affordances available to the infant is sculpted, through joint-attentional practices that reflect sociocultural norms, into a *field* of relevant solicitations. Thus, participation in patterned practices allows the installation of socially, culturally, and situationally specific expectations, which, once acquired, determine agent allocations of attention (the acquisition of abilities) and, as a result, guide action-perception.

Joint (and, eventually, shared) attentional processes (Tomasello, 2014) provide a central mechanism through which the individual is molded to conform to specific group expectations and participate in forms of cooperative action. Joint and shared attention alters the field of affordances by directing the agent to engage with specific affordances, marking them out as relevant, and making them more salient. Given the nature of the predictive hierarchy, that is, to automatically extract statistical information about the distal world in its dynamic engagement (in action-perception), the agent will encode the regularities of the solicitations that it engages (that is, the relevant affordances to which it is directed in joint and shared attention). Of course, local practices of joint and shared attention themselves depend on agents sharing sets of expectations—the same expectations that become encoded by agents as they participate in these practices. Through participation in patterned cultural practices that direct attention in specific ways, the agent acquires sets of expectations that gave rise, in the first instance, to (earlier versions of) that very form of cooperative action (see **Figure 6**). Cultural affordances are thus mediated by recursive regimes of shared attention, of which joint-attention is a special, signal case (Tomasello, 2014).

The study of everyday social interactions reveals how regimes of joint attention shape our understanding and sensory experiences of being in our worlds. For example, Goffman, who pioneered studies of face-to-face interaction in modern societies, showed how the ‘anonymized,’ ‘surface character’ of life in cities is routinized through what he called ‘civic inattention’—that is, through the many ways in which strangers avert their gazes, avoid conversations or physical contact, and reinforce private boundaries in the public sphere (Goffman, 1971, p. 385). We can follow Goffman’s lead to consider how different regimes of shared and joint-attention mediate lived experiences of meaning and being. Civic inattention, for example, is a specific regime of attention, but it is certainly not an absence of attention. In Goffman’s ‘Invisible City’ model, attentional resources are mobilized to *not* pay attention to certain features of the world, particularly other agents caught in a symbolically marked game of allegiances that renders them strange or invisible.

### Looping the Loop: Regimes of Shared Attention and Skilled Intentionality

As we have seen above, in the predictive processing scheme, attention, understood as precision-weighting of prediction error signals, is a central mechanism behind the dynamical trajectory of action-perception. The expectations about precision that guide action-perception are acquired in ontogeny and stored as high-level priors, which have the effect of arbitrating the balancing act between top–down prediction and bottom–up error signals. It follows that one pathway by which cultural affordances may be transmitted is through the manipulation of attention. This may occur in a variety of ways including what we might call ‘*regimes of shared attention.*’ In the model of affordances outlined above, this kind of attentional modulation involves carving a local field of affordances out of the larger landscape of available affordances through social practices. Local environments and their associated practices are designed to solicit particular patterns of coordinated attention from participants (Kirchhoff, 2015a; Clark, 2016). In effect, these patterns act as dynamical attractors on the field of affordances, directing action-perception in some ways rather than others (Juarrero, 1999).

In this light, one can view social norms and conventions as devices to reduce mutual uncertainty, that is, consonantly with the free-energy framework, as entropy-minimizing devices (Colombo, 2014). One must know ‘what is in the minds’ of others (such as what one would see and how one would interpret another’s action generally and in context) in order to make a successful inference (both explicit, content-involving or implicit, correlational inferences) about other agents in each situation. Goffman (1971) was hinting similar processes with his comments on the ‘faces’ we learn to perform when we interact with others in different situations. We can be a mentor in one situation and a mentee in another; a father in one and a friend in another. In Goffman’s famous comments on interaction in public, he describes (using other terms), how certain spaces afford more ‘backstage,’ ‘off-screen’ performances than others. The privacy of the home affords such relaxed ‘off-stageness,’ and the bedroom and bathroom even more so. All these instances require inferential mindreading or perspective-taking, that is, inferences about the presence or absence of other agents and their expectations as a normative guide for how one can behave. None of this depends specifically of whether these inferences consist in explicit mindreading or more implicit forms of embodied coupled enactments—both are compatible with our framework.

Now, we might suppose that the distinctly human abilities with which we are endowed result simply from better evolved predictive machinery, that is, more computationally powerful predictive hierarchies (Conway and Christiansen, 2001). However, as we argued above, in human ontogeny, it is more likely that affordances are learned through regimes of imitation, repetition, positive and negative conditioning, and culturally selective forms of attention (Meltzoff and Prinz, 2002; Whitehouse, 2002, 2004; Roepstorff and Frith, 2004; Banaji and Gelman, 2013; Veissière, 2016). The capacity for cultural learning may itself be a cultural innovation (Heyes, 2012). Indeed, the feedback or looping mechanisms between cultural practices of scaffolding individual attention (what we called regimes of attention) are themselves determined by the local ontologies (shared sets of expectations) and abilities (acquired patterns of attention and gating) of agents in that community. Repetition and reiteration of patterns of social and technological interaction, as well as reward for ‘correct’ inferences that denote an adequate grasp of relevance, prescription, and proscription (e.g., when a child ‘gets’ that some *X* means some *Y*, or figures out an ‘appropriate’ combination of meaningful elements in any given context), come to shape attentional mechanisms in ontogeny, and assist the child in successfully inferring a set of rules and categories (the culturally sanctioned sets of shared expectations).

Joint attention is usually understood as occurring in a dyad of two people, or between agents in direct interactional spheres of communication, gaze-following, finger-pointing, or other verbal or non-verbal cues (Vygotsky, 1978; Tomasello, 2014). To address more complex social situations, it is useful to revise current sociocognitive models of joint-attention to encompass fundamentally triadic situations in which ‘the third’ is the socially constituted niche of affordances, supported by local ontologies and abilities.

Shared human intentionality is sufficient to project joint attention to larger groups in the process of forming joint goals and inferring from joint expectations. Crucially, it commonly takes place without any direct interaction from members, in the many routinized, anonymous, symbolically and linguistically mediated forms of sociality, including engagement with social institutions.

To go beyond the ‘toy models’ of dyadic joint attention to grasp the process of culture transmission we need to study the dynamics of ‘designer environments’ (Goldstone et al., 2011; Salge et al., 2014). Human beings pattern their environments in a process of recursive niche construction, which in turn modulates the attributions of attention in individual agents, leading them to acquire certain sets of priors rather than others, in what Sterelny (2003) has called ‘incremental downstream epistemic engineering.’ This incremental process of constructing our own collective, epistemic niches, involves a kind of bootstrapping in which symbolically and linguistically mediated forms of human communication can be modeled as forms of re-entrant processing. Linguistically abled human beings produce patterned, structured outputs that become part of the material environment, and are subsequently picked up and further processed by other agents in ways that stabilize and elaborate a local social world (Clark, 2006, 2008). Indeed, human-constructed environments, which shape agent expectations and guide patterns of attention, can be viewed as another level of the generative statistical model of the niche, which human beings leverage to guide intelligent behavior in their sociocultural symbolically- and linguistically laden niches (Kirchhoff, 2015a; Clark, 2016). The prior knowledge that is leveraged in action-perception is thus encoded in multiple level and sites: in the hierarchical neural networks, in the organism’s phenotype (over phylogeny and ontogeny), and in patterned sociocultural practices and designer environments.

Thus, our suggestion is that regimes of attention, which mediate the acquisition of cultural affordances (both natural and conventional), are enacted through patterned practices (especially those which modulate the allocation of attention) and are embodied in sundry ways: in the predictive hierarchies of individual agents in a community, as encoded sets of expectations, and in the concrete social and cultural world, as constructed human environments, designed to solicit certain expectations and direct attention.

## Conclusion

We have outlined a framework for the study of cultural affordances in terms of neural models of predictive processing and social practices of niche construction. This approach can help account for the multilevel forms of affordance learning and transmission of affordances in socially and culturally shared regimes of joint-attention and clarify one of the central mechanisms that can explain the natural origins of semantic content. The concepts of affordance and skilled intentionality in ecological, radical embodied, and enactivist cognitive science can be supplemented with an account of the nature of affordances in the humanly constructed sociocultural niches. Turning to cultural niche construction, we argued in favor of a conception of local ontologies as sets of shared expectations acquired through the immersive engagement of the agent in feedback looping relations between shared intentionality (in the form of shared embodied expectations) and shared attention (modulated by regimes of attention). We elaborated Grice’s account of meaning by highlighting the dependence of selective responsiveness to cultural affordances on shared and joint intentionality, modes of conventionality and social normativity. We ended with an account of the patterned regimes of attention and modes of social learning that might lead to the acquisition and installation of such ontologies and affordances, leading to agent enculturation and enskillment. We hope that our proposal of a framework for the study of cultural affordances will spur further research on multilevel, recursive, nested affordances and the expectations on which they depend.

## Author Contributions

MR, SV, and LK made substantial contributions to the conception and design of the work. MR, SV, and LK drafted the work and revised it critically for important intellectual content; MR, SV, and LK have provided approval of the version to be published; MR, SV, and LK agree to be accountable for all aspects of the work in ensuring that questions related to the accuracy or integrity of any part of the work are appropriately investigated and resolved.

## Conflict of Interest Statement

The authors declare that the research was conducted in the absence of any commercial or financial relationships that could be construed as a potential conflict of interest.

## Footnotes

1 Enactive accounts reject the rigid separation of perception, cognition, and action, emphasizing that organisms cope with their environment in rolling cycles of engagement in which the distinction between action, cognition, and perception is blurred. When such a distinction is made, enactivist thinkers typically resist the traditional picture that subordinates action to perception or cognition. Theorists who draw the distinction nevertheless emphasize the deep connection between perception, cognition, and action. There are good reasons to think that action is a precondition for perception or that perception is a form of action (Clark, 2016; Kirchhoff, 2016). As we shall see in Section “The Neurodynamics of Affordances,” free-energy approaches frame perception and action as complementary ways of minimizing ‘prediction error.’ Our preference is to speak of rolling cycles of ‘action-perception’ to refer to the complex looping process whereby organisms cope with their environment. These cycles rely on various complementary computational strategies to minimize prediction error, which may (or may not) correspond to the traditional concepts of action, cognition, and perception.

2 Some might express unease at the mixed language we use, which straddles phenomenology, system dynamics, and cognitive functions. We take this as a virtue of the multilevel nature of the explanatory framework provided by the notion of affordances, which is operative at all of these different descriptive levels. Readers who would prefer to keep phenomenological description distinct from other explanatory levels (i.e., neural, social, cultural levels of explanation) can replace our talk of directly modulating the landscape or field of affordances with a more phenomenologically neutral concept, such as the organism’s ‘selective openness’ (Bruineberg and Rietveld, 2014). With this terminology, we might say that changes in the patterns of activity in the organism (states, interests, etc.) and the environment shape the organism’s selective openness to saliencies.

3 This basic cognitive formula for sociality requires three orders of automatic intentionality; that it is to say, an implicit, non-narrative, hypothesis-generating, error-reduction scenario of the “I think they think I think” variety that can be translated as “what would relevant others expect me to think/feel/do in this situation?”

4 The notion of *variation* and improvisation in action within a convention is very important. Humans do not simply obey prescribed expectations, but also resist, transgress and transform them. Specific fields of joint-intentional affordances, thus, invariably entail different licenses for improvisation on expected behavior. The background formula for action is not simply “what would others expect me to do here?” but also “how much license or room to improvise do I have here given what the set of local cues tells me about others’ expectations and the norms that should otherwise govern my behavior in this specific situation?”

5 We should note a few limitations of the ‘epidemic’ metaphor: (i) representations are not merely transmitted through contagion, but through many different means, modes of communication, and practices that are themselves culturally mediated; (ii) they reside not just in individuals, but also in artifacts and institutions; and (iii) they are usually not simply replicated, but modified or transformed by each individual or institution that takes them up.

6 ‘Teleosemiotics’ is teleosemantics minus the semantics, that is, using the teleosemantic framework developed by Millikan (1984, 2004, 2005) to explain how organisms develop selective intentional response tendencies without trying to provide thereby an account of semantic content (Hutto and Myin, 2013). See also Kiverstein and Rietveld (2015) for a complementary account of minimal intentionality as a contentless form of skilled intentionality.
